# Pharmacological activation of SIRT1 alleviates sepsis-associated acute kidney injury by improving renal mitochondrial energy metabolism

**DOI:** 10.1080/0886022X.2026.2723540

**Published:** 2026-09-10

**Authors:** Rui Zhu, Hairong Yang, Li Liu, Yuanyuan Gao, Xiaofang Gao

**Affiliations:** ICU, People’s Hospital of Ningxia Hui Autonomous Region, Ningxia Medical University, Yinchuan, Ningxia, China

**Keywords:** Sepsis-associated acute kidney injury, silent information regulator 1, Mitochondrial, energy metabolism

## Abstract

Sepsis-associated acute kidney injury (SA-AKI) is a frequent and severe complication of sepsis and is closely associated with increased mortality. Mitochondrial dysfunction and impaired energy metabolism are important contributors to SA-AKI pathogenesis. Silent information regulator 1 (SIRT1), a nicotinamide adenine dinucleotide (NAD^+^)-dependent deacetylase, regulates cellular metabolism, oxidative stress, and mitochondrial homeostasis. However, its role in septic renal mitochondrial dysfunction remains incompletely understood. In this study, an LPS-induced NRK-52E rat kidney epithelial cell injury model and a cecal ligation and puncture (CLP)-induced sepsis rat model were established. SIRT1-related signaling was pharmacologically modulated using the SIRT1 activator SRT1720 or the SIRT1 inhibitor EX-527. Cell viability, SIRT1 mRNA and protein abundance, mitochondrial ultrastructure, oxidative stress, mitochondrial membrane potential, ATP content, ATPase activity, and non-esterified fatty acid levels were assessed. LPS exposure reduced SIRT1 expression in NRK-52E cells and was accompanied by decreased cell viability, mitochondrial structural damage, oxidative stress, and impaired energy metabolism. SRT1720 attenuated these changes, whereas EX-527 aggravated them. Similarly, in CLP-induced septic rats, renal SIRT1 expression was decreased, together with renal injury and mitochondrial metabolic dysfunction. SRT1720 ameliorated renal pathological injury and mitochondrial-related abnormalities, whereas EX-527 worsened these changes. These findings suggest that SIRT1 activation attenuates SA-AKI, at least partly by maintaining renal mitochondrial energy homeostasis.

## Introduction

Sepsis is a life threatening syndrome characterized by organ dysfunction resulting from a dysregulated host response to infection and remains a leading cause of morbidity and mortality in critically ill patients worldwide [1]. Despite advances in antimicrobial therapy and supportive care [2], sepsis continues to be associated with a high incidence of multiple organ dysfunction, among which acute kidney injury (AKI) is one of the most common and devastating complications [3]. Sepsis-associated acute kidney injury (SA-AKI) occurs in a substantial proportion of septic patients and is strongly correlated with increased mortality [4], prolonged intensive care unit stay, and poor long-term renal outcomes.

The pathophysiology of SA-AKI is complex and differs from that of ischemic or nephrotoxic AKI [5]. Traditional concepts attributing renal injury primarily to reduced renal perfusion are insufficient to fully explain the development of SA-AKI. Clinical and experimental studies have shown that SA-AKI can occur even in the presence of preserved or only mildly reduced renal blood flow. Increasing evidence indicates that renal microcirculatory dysfunction, inflammatory and immune dysregulation, and metabolic disturbances collectively contribute to renal injury during sepsis [6].

Mitochondria are central regulators of cellular energy metabolism, redox balance, and cell survival, and their dysfunction has emerged as a key mechanism in the development of SA-AKI [7]. During sepsis, excessive production of reactive oxygen species (ROS), loss of mitochondrial membrane potential, impaired oxidative phosphorylation, and reduced ATP synthesis lead to cellular energy deficiency and oxidative stress [8]. These alterations disrupt tubular epithelial cell function and may ultimately result in cell injury or death. Therefore, mitochondrial energy metabolic dysfunction is increasingly recognized as a critical contributor to the initiation and progression of SA-AKI.

Silent information regulator 1 (SIRT1), a nicotinamide adenine dinucleotide (NAD^+^)-dependent deacetylase, is an important regulator of cellular metabolism, oxidative stress, and mitochondrial function [9]. SIRT1 has been reported to participate in mitochondrial biogenesis, antioxidant defense, and energy homeostasis in various tissues, and accumulating evidence suggests that it plays a protective role in metabolic stress-related diseases [10]. However, its involvement in sepsis-induced renal mitochondrial energy metabolic dysfunction remains incompletely understood. In particular, whether SIRT1 modulates mitochondrial structure, oxidative stress, and energy metabolism during SA-AKI has not been fully clarified.

Based on these considerations, the present study aimed to determine whether pharmacological modulation of SIRT1 affects mitochondrial dysfunction and renal injury under septic conditions. Using an LPS-induced NRK-52E rat kidney epithelial cell injury model and a cecal ligation and puncture (CLP)-induced sepsis rat model, we examined the effects of the SIRT1 activator SRT1720 and the SIRT1 inhibitor EX-527 on SIRT1 expression, mitochondrial ultrastructure, oxidative stress, mitochondrial membrane potential, ATP production, ATPase activity, and renal injury. This study provides experimental evidence for the involvement of SIRT1-related signaling in mitochondrial energy metabolic dysfunction during SA-AKI and supports SIRT1 as a potential therapeutic target for septic renal injury.

## Materials and methods

### Main reagents

The main reagents used in this study were as follows: lipopolysaccharide (Abmole, Cat. No. M9524), SRT1720 (Abmole, Cat. No. M11030), EX-527 (Abmole, Cat. No. M1708), Cell Counting Kit-8 (Abmole, Cat. No. M4839), TRIzon Reagent (Cwbio, Cat. No. CW0580), RTIII All-in-One Mix reverse transcription kit (Monad, Cat. No. MR05101), SYBR Green qPCR Mix (Monad, Cat. No. MQ10101), RIPA lysis buffer (Solarbio, Cat. No. R0010), BCA protein assay kit (Cwbio, Cat. No. CW0014), anti-SIRT1 antibody (Boster, Cat. No. PB0173), anti-β-actin antibody (Proteintech, Cat. No. 66009-1-Ig), mitochondrial ROS detection reagent (Beyotime, Cat. No. S0033S), JC-1 staining working solution (Solarbio, Cat. No. M8650), superoxide dismutase (SOD) assay kit (Jianglai Biology, Cat. No. JL-T0781), malondialdehyde (MDA) assay kit (Jianglai Biology, Cat. No. JL-T0761), ATP assay kit (Jianglai Biology, Cat. No. JL-T0632), Ca2^+^/Mg2^+^-ATPase assay kit (Jianglai Biology, Cat. No. JL-T0638), Ca2^+^-ATPase assay kit (Jianglai Biology, Cat. No. JL-T0640), Na^+^/K^+^-ATPase assay kit (Jianglai Biology, Cat. No. JL-T0658), free fatty acid assay kit (Jianglai Biology, Cat. No. JL-T1312), creatinine assay kit (Jianglai Biology, Cat. No. JL-T0928), and urea nitrogen assay kit (Jianglai Biology, Cat. No. JL-T1013).

### Cell culture and treatments

The rat renal tubular epithelial cell line NRK-52E was obtained from Guangzhou Keluojie Biotechnology Co., Ltd. (Guangzhou, China). Cells were maintained in DMEM supplemented with 10% fetal bovine serum and 1% penicillin-streptomycin at 37 °C in a humidified incubator with 5% CO_2_.

To establish an *in vitro* sepsis related injury model, NRK-52E cells were exposed to LPS for 24 h. Based on preliminary dose response experiments, 30 μg/mL LPS was selected for subsequent assays. To pharmacologically modulate SIRT1 related signaling, cells were treated with the SIRT1 activator SRT1720 or SIRT1 inhibitor EX-527. Cells were pretreated with SRT1720 (5 μM) or EX-527 (200 nM) for 1 h before LPS stimulation and then incubated for 24 h. RT-qPCR and Western blot analyses in the present study were used to assess treatment-associated changes in SIRT1 mRNA and protein abundance. Cells were assigned to four groups: Control, LPS, LPS + SRT1720, and LPS + EX-527.

### Cell viability assay

Cell viability was evaluated using CCK-8 according to the manufacturer’s instructions. Briefly, NRK-52E cells were seeded into 96-well plates and subjected to the indicated treatments. After treatment, 10 μL of CCK-8 reagent was added to each well and incubated for 2 h at 37 °C. Absorbance was measured at 450 nm using a microplate reader.

### RNA extraction and RT-qPCR

RT-qPCR was performed to determine the mRNA expression level of SIRT1 in cultured cells and renal tissues. Total RNA was extracted from cultured cells or renal tissues using TRIzon Reagent according to the manufacturer’s instructions. Complementary DNA (cDNA) was synthesized from 1 μg total RNA using the RTIII All-in-One Mix reverse transcription kit. Quantitative real-time PCR was performed using SYBR Green qPCR Mix on a real-time PCR system. The thermal cycling conditions were as follows: initial denaturation at 95 °C for 30 s, followed by 40 cycles of 95 °C for 5 s and 60 °C for 30 s. Relative SIRT1 mRNA expression was calculated using the 2^−ΔΔCt^ method, with β-actin as the internal reference gene. The primer sequences used in this study were as follows:

SIRT1 forward, 5′-GCAAAGAAGAAACAGCATTGAAGC-3′;
SIRT1 reverse, 5′-GCTGTTGCAAAGGAACCATGA-3′;
β-actin forward, 5′-GCTGTGCTATGTTGCCCTAGACTTC-3′;
β-actin reverse, 5′-GGAACCGCTCATTGCCGATAGTG-3′.

### Western blot analysis

Total protein was extracted from cells or renal tissues using RIPA lysis buffer supplemented with protease inhibitor. Protein concentrations were determined using a BCA protein assay kit. Equal amounts of protein (30 μg per lane) were separated by SDS-PAGE and transferred onto PVDF membranes. After blocking with 5% nonfat milk in TBST for 1 h at room temperature, membranes were incubated overnight at 4 °C with anti-SIRT1 antibody (1:1000 for Western blot) and anti-β-actin antibody (1:40,000). After washing, membranes were incubated with HRP-conjugated secondary antibody for 1 h at room temperature. Protein bands were visualized using an enhanced chemiluminescence substrate and quantified using ImageJ software. Protein expression levels were normalized to β-actin.

### Transmission electron microscopy

Fresh kidney tissues were rapidly collected from the target region within 1–3 min after sacrifice and cut into approximately 1 mm³ tissue blocks. The tissue blocks were immediately immersed in electron microscopy fixative and stored at 4 °C in the dark before further processing.

After primary fixation, samples were rinsed three times with PBS for 15 min each and post-fixed with 1% osmium tetroxide prepared in 0.1 M PB for 2 h at room temperature in the dark. The samples were then washed again with 0.1 M PBS and dehydrated through a graded ethanol series of 30%, 50%, 70%, 80%, 95%, and 100%, followed by dehydration in 100% acetone. After dehydration, tissues were infiltrated with a mixture of acetone and 812 embedding resin, followed by pure 812 resin infiltration and embedding. The resin blocks were polymerized at 60 °C for 48 h.

Semi-thin sections of 1.5 μm were prepared and stained with toluidine blue for localization under light microscopy. Ultrathin sections of 60–80 nm were then cut using an ultramicrotome and collected on 150-mesh copper grids. The sections were stained with 2% uranyl acetate in ethanol for 8 min in the dark, followed by lead citrate staining for 8 min while avoiding carbon dioxide exposure. After washing with ultrapure water and drying overnight at room temperature, the sections were observed and imaged using a transmission electron microscope.

### Assessment of oxidative stress, mitochondrial function, and energy metabolism

For renal mitochondrial ROS detection, fresh kidney tissues were used to isolate mitochondria according to the manufacturer’s instructions. The mitochondrial suspension was incubated with ROS working solution in the dark for 20 min, and fluorescence intensity was measured using a fluorescence microplate reader. The results were normalized to protein concentration.

Mitochondrial membrane potential in renal tissues was assessed by JC-1 staining. Fresh kidney tissues were digested with collagenase to prepare single-cell suspensions, incubated with JC-1 working solution for 20 min in the dark, washed with PBS, and analyzed by flow cytometry. JC-1 fluorescence intensity was used as an indicator of mitochondrial membrane potential.

Intracellular ROS levels in NRK-52E cells were detected using DCFH-DA staining. After treatment, cells were incubated with 10 μmol/L DCFH-DA at 37 °C for 20 min in the dark, washed with PBS, collected, and analyzed by flow cytometry. Mitochondrial membrane potential in NRK-52E cells was evaluated using JC-1 staining followed by flow cytometric analysis, and the red/green fluorescence ratio was calculated.

Cell viability was measured using the CCK-8 assay. NRK-52E cells were seeded into 96-well plates, treated with different concentrations of LPS for 24 h, and then incubated with CCK-8 solution for 2 h. Absorbance was measured at 450 nm.

SOD activity, MDA content, ATP content, Ca^2+^/Mg^2+^-ATPase activity, Ca^2+^-ATPase activity, Na^+^/K^+^-ATPase activity, and NEFA levels were measured using corresponding commercial assay kits according to the manufacturers’ instructions. For tissue samples, the results were normalized to protein concentration when appropriate.

### Animals and experimental protocol

To minimize variability related to sex, age, body weight, and housing conditions, male Sprague-Dawley rats aged 6–8 weeks were used, and all animals were maintained under specific pathogen-free conditions before randomization. Rats were obtained from Beijing Speford Biotechnology Co., Ltd., with animal production license no. SCXK (Jing) 2024-0001. A total of 16 rats were randomly assigned to four groups, with four rats in each group: Sham, CLP, CLP+SRT1720, and CLP+EX-527. All experimental procedures were reviewed and approved by the Institutional Animal Care and Use Committee of People’s Hospital of Ningxia Hui Autonomous Region under approval no. [2023]-NZR-222 and were performed in accordance with institutional guidelines for the care and use of laboratory animals.

The doses of SRT1720 and EX-527 were selected based on previous studies [11,12]. SRT1720 (20 mg/kg) or EX-527 (5 mg/kg) was administered intraperitoneally 4 h before CLP surgery. Sepsis was induced by cecal ligation and puncture (CLP)[11]. Briefly, rats were anesthetized with sodium pentobarbital (50 mg/kg, intraperitoneally), and the abdomen was shaved and disinfected. A 2-cm midline abdominal incision was made under aseptic conditions to expose the cecum with minimal manipulation. The cecum was ligated with 4-0 silk sutures at approximately 50% of its length distal to the ileocecal valve, taking care not to obstruct intestinal continuity. The ligated cecum was then punctured twice with an 18-gauge needle at two separate sites, and a small amount of fecal material was gently extruded to ensure patency. The cecum was returned to the abdominal cavity, and the abdominal wall and skin were closed in layers. After surgery, rats received sterile normal saline for fluid resuscitation. Sham-operated rats underwent the same laparotomy and cecum exposure procedures, but the cecum was neither ligated nor punctured.

Animal health and behavior were monitored every 4 h during the 24-h observation period after CLP or sham surgery. Monitoring included general appearance, activity, posture, response to stimulation, respiratory status, mobility, food and water intake, wound condition, and signs of pain or distress.

The experiment lasted for 24 h after CLP or sham surgery. At the scheduled endpoint 24 h after surgery, all surviving animals were euthanized by overdose of sodium pentobarbital, and blood and kidney tissues were collected for subsequent analyses. In this study, a total of 16 rats were used, all 16 rats were euthanized at the scheduled endpoint, and no animals were found dead before the scheduled endpoint or before meeting humane endpoint criteria. Therefore, all animals underwent planned euthanasia with an overdose of sodium pentobarbital in accordance with the approved protocol.

### Histological and immunohistochemical evaluation

Renal tissues were fixed in 10% neutral-buffered formalin, embedded in paraffin, and sectioned at 4 μm. For hematoxylin and eosin (H&E) staining, paraffin sections were deparaffinized in dewaxing solution, rehydrated through graded ethanol, and rinsed with running tap water. The sections were stained with hematoxylin for 3 min, differentiated for several seconds, blued, and then counterstained with eosin. After dehydration through graded ethanol and clearing, the sections were mounted with neutral resin. The stained sections were scanned using a digital slide scanner, and renal histopathological changes were evaluated under light microscopy by investigators blinded to the experimental groups.

Renal tubular injury was assessed based on tubular epithelial cell swelling, tubular dilation, tubular necrosis, cast formation, inflammatory cell infiltration, and structural disruption. Tubular injury was scored according to the percentage of damaged renal tubules as follows: 0, no obvious injury; 1, <10% injured tubules; 2, 10-25% injured tubules; 3, 26-50% injured tubules; and 4, >50% injured tubules. Several non-overlapping fields were evaluated for each section, and the average score was used for statistical analysis.

For immunohistochemistry, paraffin-embedded kidney sections were deparaffinized, rehydrated, and subjected to antigen retrieval in citrate buffer (pH 6.0). After natural cooling, the sections were washed with PBS. Endogenous peroxidase activity was blocked with 3% hydrogen peroxide for 25 min at room temperature in the dark. After washing with PBS, nonspecific binding was blocked with 3% BSA for 30 min at room temperature. The sections were then incubated with anti-SIRT1 primary antibody (1:50) overnight at 4 °C in a humidified chamber. After washing, the sections were incubated with the corresponding HRP-conjugated secondary antibody at room temperature, followed by visualization with DAB substrate. The reaction was monitored under a microscope and stopped when brown-yellow positive staining appeared. Finally, the sections were counterstained with hematoxylin, dehydrated, cleared, and mounted.

Immunohistochemical staining was evaluated under the same imaging conditions. Representative non-overlapping fields were selected from each section, and positive staining was quantified as average optical density (AOD) using image analysis software. All histopathological and immunohistochemical analyses were performed by investigators blinded to the experimental groups.

### Renal function assessment

Blood samples were collected at the time of sacrifice and centrifuged to obtain serum. Serum creatinine and blood urea nitrogen (BUN) levels were measured using an automatic biochemical analyzer (BS-240VET; Shenzhen Mindray Bio-Medical Electronics Co., Ltd., Shenzhen, China) according to the manufacturer’s instructions.

### Statistical analysis

All data are presented as mean ± standard deviation (SD). Statistical analyses were performed using SPSS 27.0 and GraphPad Prism 10.6.0. Comparisons among multiple groups were performed using one-way analysis of variance (ANOVA), followed by Tukey’s *post hoc* test for multiple comparisons. A two-tailed P value < 0.05 was considered statistically significant.

## Results

### Establishment of the LPS-induced NRK-52E cell injury model and selection of treatment conditions

**Figure 1.**
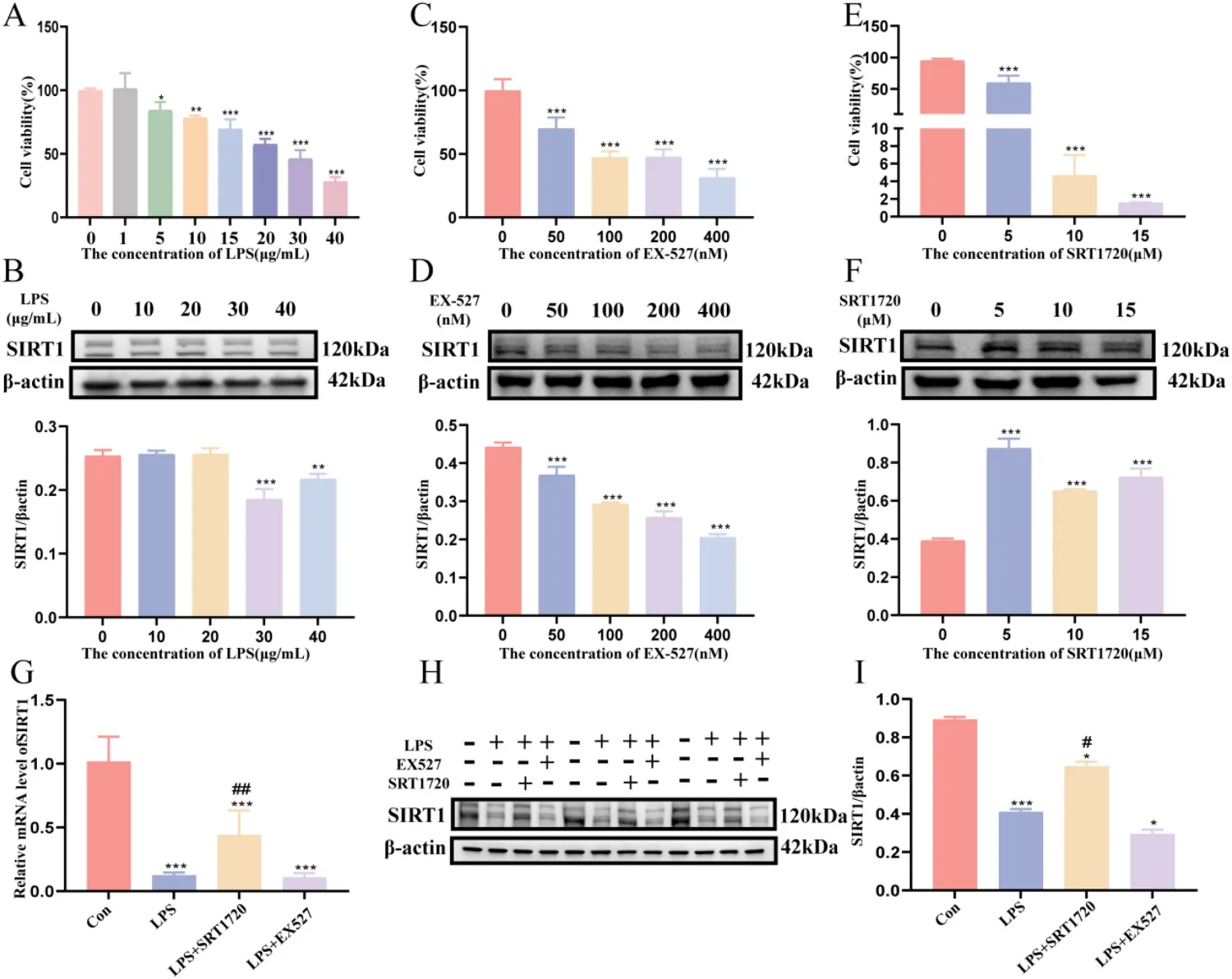
Establishment of the LPS-induced NRK-52E cell injury model and pharmacological modulation of SIRT1. (A) Cell viability after 24h exposure to different concentrations of LPS. (B) SIRT1 protein expression after LPS treatment. (C, D) Effects of EX-527 on cell viability and SIRT1 protein expression. (E, F) Effects of SRT1720 on cell viability and SIRT1 protein expression. (G) SIRT1 mRNA expression. (H, I) Representative Western blot bands and quantification of SIRT1 protein expression. Data are presented as mean ± SD (*n* = 3). *p < 0.05 vs control, ^#^*p* < 0.05 vs LPS.

To establish an *in vitro* model of sepsis-related renal tubular epithelial cell injury, NRK-52E cells were exposed to increasing concentrations of LPS for 24 h. CCK-8 analysis showed that LPS treatment caused a concentration-dependent reduction in cell viability (Figure 1A). Western blot analysis further showed that LPS exposure was associated with decreased SIRT1 protein abundance in NRK-52E cells (Figure 1B). At 30 μg/mL LPS, cell viability was markedly reduced and SIRT1 protein abundance reached the lowest level among the tested concentrations. Therefore, this concentration was selected for subsequent experiments.

To determine the working concentration of EX-527, NRK-52E cells were treated with increasing concentrations of the compound for 24 h. CCK-8 analysis showed a concentration-dependent reduction in cell viability (Figure 1C), and Western blot analysis demonstrated a corresponding decrease in SIRT1 protein abundance (Figure 1D). Based on these findings, 200 nM EX-527 was selected for further experiments.

NRK-52E cells were also treated with different concentrations of SRT1720. Among the tested concentrations, 5 μM SRT1720 maintained the highest cell viability and induced the greatest increase in SIRT1 protein expression (Figure 1E and F), whereas higher concentrations markedly reduced cell viability. Therefore, 5 μM SRT1720 was selected for subsequent experiments.

Based on these conditions, NRK-52E cells were divided into four groups: Control, LPS, LPS + SRT1720, and LPS + EX-527. RT-qPCR analysis showed that SIRT1 mRNA expression was significantly decreased in the LPS group compared with the control group (Figure 1G). Western blot analysis showed a similar reduction in SIRT1 protein abundance (Figure 1H and I). Compared with the LPS group, treatment with EX-527 was associated with further reductions in SIRT1 mRNA and protein abundance, whereas treatment with SRT1720 was associated with partial restoration of SIRT1 mRNA and protein abundance (Figure 1G–I).

### Effects of pharmacological modulation of SIRT1 related signaling on LPS-induced NRK-52E cell injury

**Figure 2.**
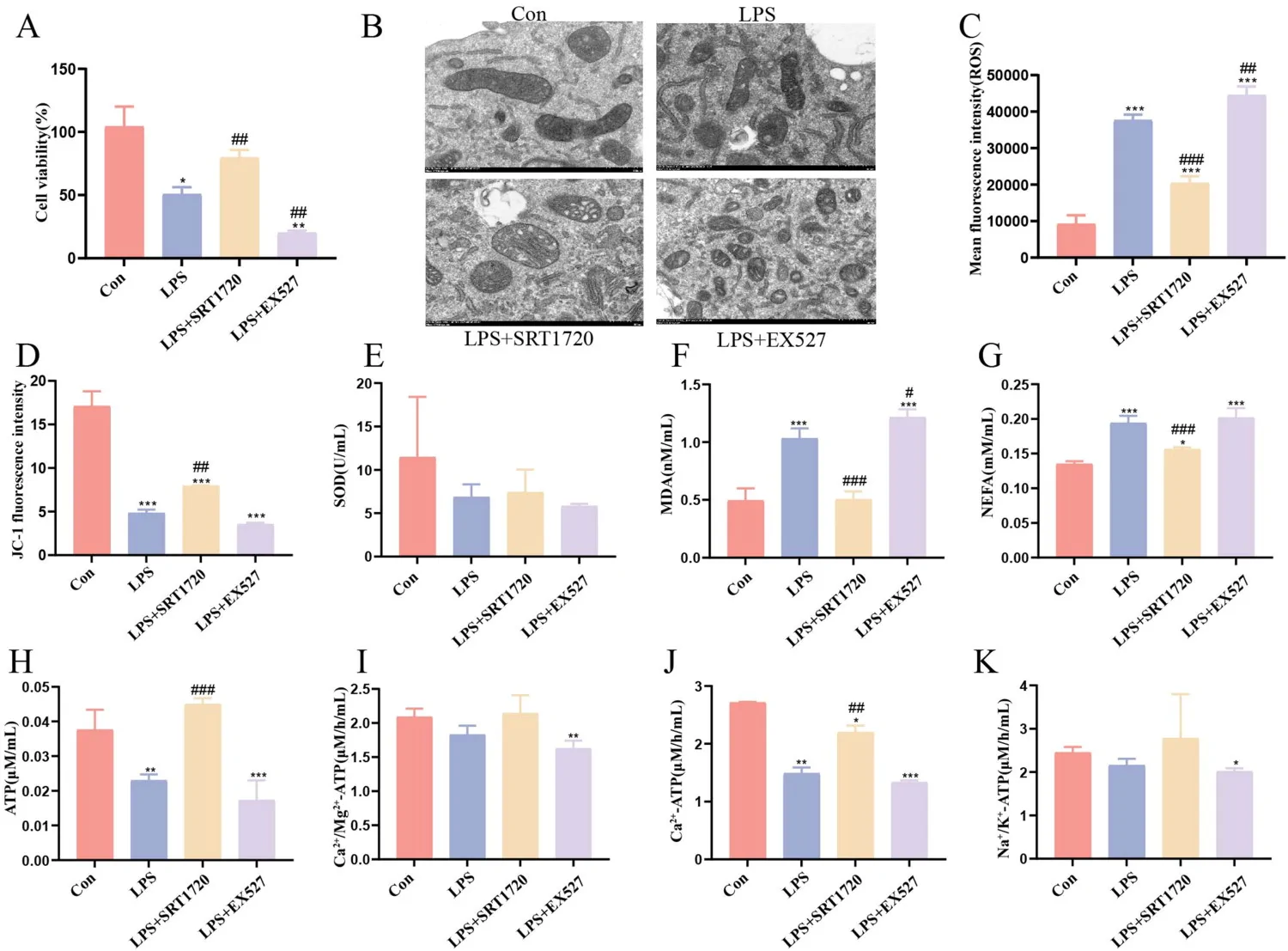
Effects of SIRT1 modulation on LPS-induced NRK-52E cell injury and mitochondrial dysfunction. (A) Cell viability measured by CCK-8 assay. (B) Representative TEM images showing mitochondrial ultrastructure. (C) Mitochondrial ROS levels. (D) Mitochondrial membrane potential assessed by JC-1 staining. (E, F) SOD and MDA levels. (G) NEFA levels. (H) ATP content. (I–K) Ca²⁺/Mg²⁺-ATPase, Ca²⁺-ATPase, and Na⁺/K⁺-ATPase activities. Data are presented as mean ± SD (*n* = 3). *p < 0.05 vs control, ^#^*p* < 0.05 vs LPS.

CCK-8 analysis showed that cell viability was significantly reduced in the LPS group compared with the control group (Figure 2A). Compared with the LPS group, SRT1720 treatment significantly improved cell viability, whereas EX-527 treatment further reduced viability (Figure 2A).

Transmission electron microscopy showed that NRK-52E cells in the control group exhibited relatively intact mitochondrial ultrastructure with well-organized cristae. In contrast, LPS stimulation induced pronounced mitochondrial damage, including mitochondrial swelling, cristae disruption or reduction, matrix loosening, and vacuolar degeneration (Figure 2B). Compared with the LPS group, SRT1720 treatment attenuated mitochondrial swelling and ultrastructural damage, whereas EX-527 further aggravated mitochondrial injury (Figure 2B). These findings suggest that activation of SIRT1-related signaling protects against LPS-induced renal tubular epithelial cell injury, while SIRT1 inhibition exacerbates mitochondrial structural damage.

### Effects of pharmacological modulation of SIRT1 related signaling on mitochondrial function and energy metabolism in NRK-52E cells

Compared with the control group, LPS exposure significantly increased mitochondrial ROS levels (Figure 2C) and decreased mitochondrial membrane potential (Figure 2D). In addition, MDA levels (Figure 2F) and NEFA levels (Figure 2G) were significantly increased, whereas ATP content was markedly reduced (Figure 2H). SOD activity showed a downward trend in the LPS group, although the difference did not reach statistical significance (Figure 2E). Among the ATPase-related indices, Ca2^+^-ATPase activity was significantly decreased (Figure 2J), whereas Ca2^+^/Mg2^+^-ATPase (Figure 2I) and Na^+^/K^+^-ATPase activities (Figure 2K) showed downward trends.

Compared with the LPS group, treatment with SRT1720 significantly reduced mitochondrial ROS production (Figure 2C), restored mitochondrial membrane potential (Figure 2D), and decreased MDA levels (Figure 2F). SRT1720 also increased ATP content (Figure 2H), improved Ca2^+^-ATPase activity (Figure 2J), and reduced NEFA levels (Figure 2G). In addition, SOD activity (Figure 2E), Ca2^+^/Mg2^+^-ATPase activity (Figure 2I), and Na^+^/K^+^-ATPase activity (Figure 2K) showed a tendency toward improvement after SRT1720 treatment, although some of these changes did not reach statistical significance. In contrast, EX-527 treatment further aggravated mitochondrial dysfunction and metabolic impairment, as reflected by higher mitochondrial ROS and MDA levels, lower mitochondrial membrane potential and ATP content, and higher NEFA levels compared with the LPS group (Figure 2C–H).

### Pharmacological modulation of SIRT1 altered renal SIRT1 expression in septic rats

**Figure 3.**
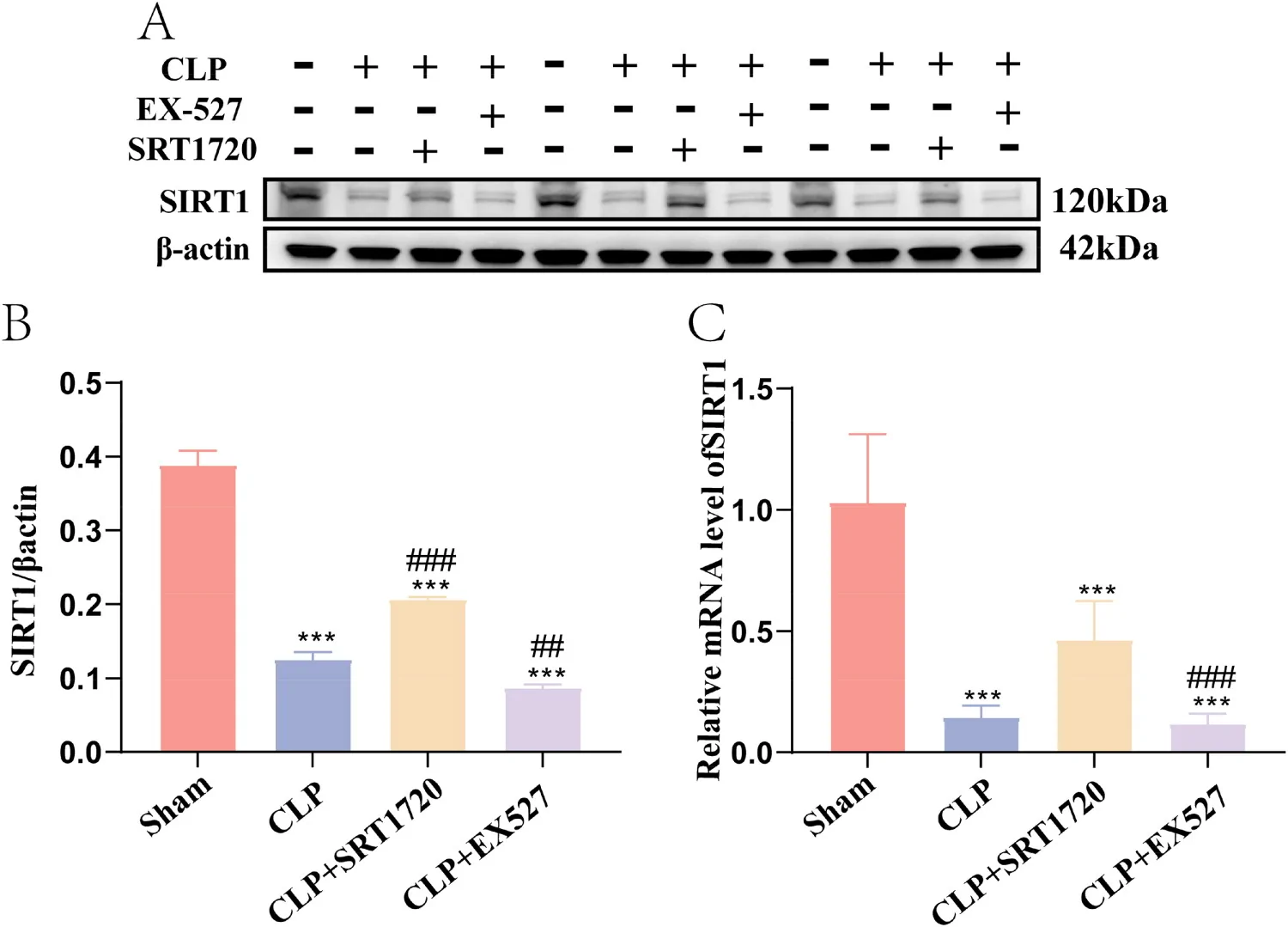
Effects of SIRT1 modulation on renal SIRT1 expression in septic rats. (A) Representative immunoblots of SIRT1 in renal tissues. (B) Densitometric quantification of SIRT1 protein expression. (C) Relative SIRT1 mRNA expression determined by RT-qPCR. Data are presented as mean ± SD (*n* = 4). *p < 0.05 vs. Sham, ^#^*p* < 0.05 vs. CLP.

To determine whether septic injury was associated with altered renal SIRT1 expression *in vivo*, a CLP-induced sepsis rat model was established. RT-qPCR analysis showed that renal SIRT1 mRNA expression was significantly decreased in the CLP group compared with the sham group (Figure 3C). Western blot analysis demonstrated a corresponding reduction in renal SIRT1 protein abundance (Figure 3A and ^B^).

Compared with the CLP group, treatment with EX-527 was associated with further reductions in renal SIRT1 mRNA and protein abundance, whereas treatment with SRT1720 was associated with increased renal SIRT1 mRNA and protein abundance (Figure 3A–C).

### Effects of pharmacological modulation of SIRT1-related signaling on renal injury and mitochondrial dysfunction in septic rats

**Figure 4.**
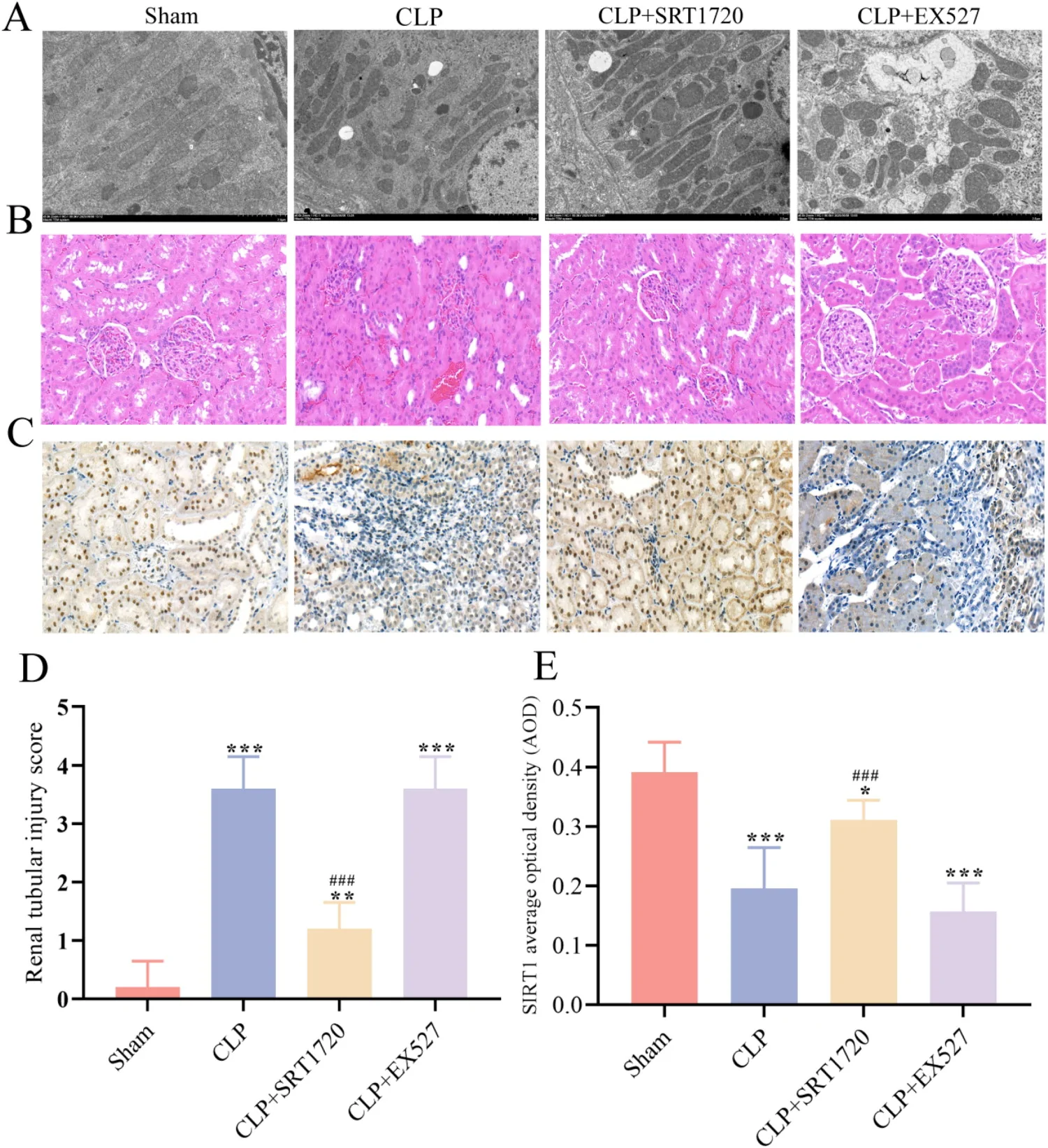
Effects of SIRT1 modulation on renal ultrastructure, histopathological injury, and SIRT1 expression in septic rats. (A) Representative transmission electron microscopy images. (B) Representative H&E-stained images of renal tissues. (C) Representative immunohistochemical staining images of SIRT1 in renal tissues. (D) Quantification of renal tubular injury scores. (E) Quantification of SIRT1 immunohistochemical staining. Data are presented as mean ± SD (*n* = 4). *p < 0.05 vs. Sham, ^#^*p* < 0.05 vs. CLP.

Transmission electron microscopy showed that the Sham group exhibited relatively intact mitochondrial morphology with well-preserved cristae in rat kidney epithelial cells. In contrast, the CLP group displayed marked mitochondrial swelling, cristae disruption, and ultrastructural damage. These mitochondrial abnormalities were attenuated in the CLP+SRT1720 group, whereas more severe mitochondrial structural injury was observed in the CLP+EX-527 group (Figure 4A).

Hematoxylin and eosin staining demonstrated obvious renal histopathological injury in the CLP group, characterized by tubular epithelial swelling, tubular structural disorganization, luminal dilation, and inflammatory injury. These pathological changes were alleviated by SRT1720 treatment but remained severe in the CLP+EX-527 group (Figure 4B). Consistently, the renal tubular injury score was significantly increased in the CLP group compared with the Sham group. SRT1720 treatment markedly reduced the tubular injury score, whereas EX-527 treatment failed to alleviate CLP-induced tubular damage and maintained a high injury score (Figure 4D).

Immunohistochemical staining showed that renal SIRT1 expression was markedly decreased in the CLP group compared with the Sham group. SRT1720 treatment increased SIRT1 staining intensity, whereas EX-527 treatment further reduced SIRT1 expression (Figure 4C). Quantitative analysis of average optical density (AOD) confirmed that SIRT1 expression was significantly reduced after CLP surgery, partially restored by SRT1720 treatment, and further decreased by EX-527 treatment (Figure 4E). These findings suggest that SIRT1 activation attenuates CLP-induced mitochondrial dysfunction and renal histopathological injury, whereas SIRT1 inhibition aggravates renal damage in septic rats.

### Effects of pharmacological modulation of SIRT1-related signaling on renal function, oxidative stress, and mitochondrial energy metabolism in septic rats

**Figure 5.**
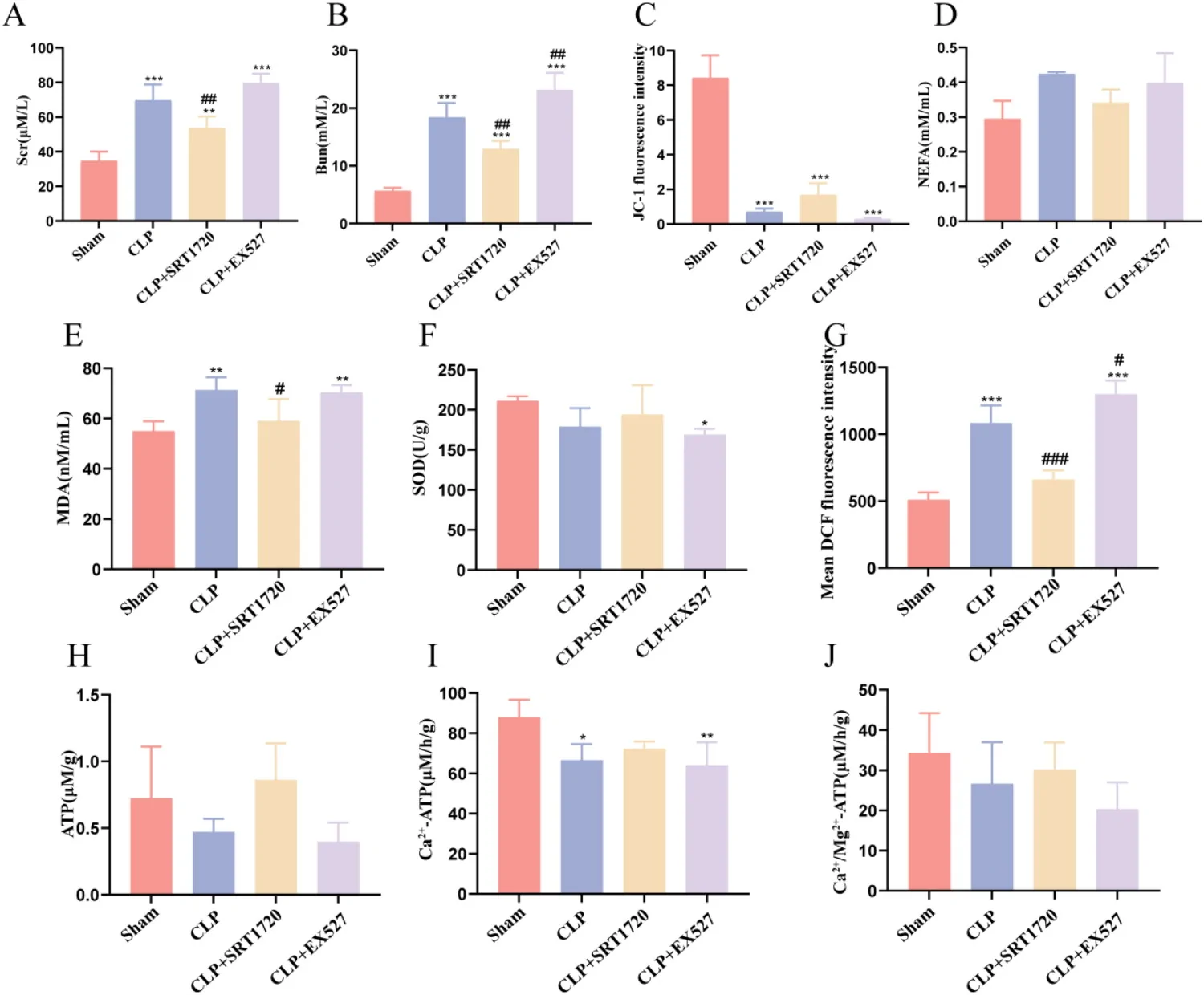
Effects of SIRT1 modulation on renal function, oxidative stress, and mitochondrial energy metabolism in septic rats. (A, B) Serum creatinine (SCr) and urea nitrogen (BUN) levels. (C) Mitochondrial membrane potential (ΔΨm) in renal tissues by JC-1 fluorescence intensity. (D) Serum non-esterified fatty acid (NEFA) levels. (E–G) Renal MDA levels, SOD activity, and ROS levels. (H) Renal ATP content. (I, J) Renal Ca²⁺-ATPase and Ca²⁺/Mg²⁺-ATPase activities. Data are presented as mean ± SD (*n* = 4).*p < 0.05 vs. Sham, ^#^*p* < 0.05 vs. CLP.

Compared with the Sham group, the CLP group showed significantly increased serum creatinine and BUN levels, indicating impaired renal function after sepsis induction. SRT1720 treatment significantly reduced serum creatinine and BUN levels compared with the CLP group, whereas EX-527 treatment failed to improve renal dysfunction and maintained high levels of these renal function markers (Figure 5A and ^B^).

Mitochondrial functional analysis showed that the CLP group exhibited a marked decrease in mitochondrial membrane potential, as indicated by reduced JC-1 fluorescence intensity, together with increased ROS production and elevated MDA levels (Figure 5C, E, G). These findings suggest that CLP-induced septic kidney injury was accompanied by mitochondrial dysfunction and oxidative stress. SRT1720 treatment partially restored mitochondrial membrane potential and significantly reduced ROS and MDA levels compared with the CLP group, whereas EX-527 treatment aggravated these abnormalities (Figure 5C, E, G).

In addition, Ca^2+^-ATPase activity was significantly reduced in the CLP group compared with the Sham group, suggesting impaired mitochondrial energy-related enzymatic function. SRT1720 treatment showed a tendency to improve Ca^2+^-ATPase activity, whereas EX-527 treatment maintained a reduced level (Figure 5I). However, no statistically significant differences were observed among the groups with respect to NEFA levels, ATP content, or Ca^2+^/Mg^2+^-ATPase activity, although numerical trends were present (Figure 5D, H, J). SOD activity also showed a decreasing trend after CLP and a partial recovery after SRT1720 treatment, but the differences were not statistically significant in the current dataset (Figure 5F).

Together, these results indicate that pharmacological activation of SIRT1 improves renal function and attenuates oxidative stress and mitochondrial dysfunction in septic rats, whereas SIRT1 inhibition aggravates these pathological changes. Nevertheless, the ATP, NEFA, SOD, and Ca^2+^/Mg^2+^-ATPase results should be interpreted cautiously because of the relatively large variability and lack of statistical significance in some comparisons.

## Discussion

Sepsis-associated acute kidney injury (SA-AKI) is not solely explained by renal hypoperfusion or ischemic injury. Recent studies have shown that inflammatory injury, microcirculatory disturbance, oxidative stress, and metabolic dysregulation jointly contribute to renal dysfunction during sepsis [13,14]. Among these mechanisms, mitochondrial dysfunction is particularly important because renal tubular cells depend heavily on mitochondrial oxidative phosphorylation to maintain ATP-dependent transport and epithelial polarity. Impaired mitochondrial function during sepsis can lead to excessive ROS production, loss of mitochondrial membrane potential, reduced ATP generation, and subsequent tubular dysfunction [15,16]. In the present study, SIRT1 expression was decreased in both LPS treated NRK-52E rat kidney epithelial cells and CLP-induced septic rat kidneys. This reduction was accompanied by mitochondrial structural damage, increased oxidative stress, impaired mitochondrial membrane potential, disturbed ATP-related metabolic activity, and aggravated renal injury. Pharmacological activation of SIRT1 by SRT1720 alleviated these abnormalities, whereas inhibition of SIRT1 by EX-527 generally worsened mitochondrial dysfunction and renal damage. These findings suggest that reduced SIRT1 activity may contribute to mitochondrial energy metabolic dysfunction in SA-AKI and that restoration of SIRT1-related signaling may help preserve mitochondrial homeostasis and renal function during sepsis.

SIRT1 is a NAD^+^-dependent deacetylase that integrates cellular redox state with transcriptional and post-translational control of metabolism, stress resistance, and inflammation [17]. In kidney disease, SIRT1 has been implicated in multiple injury contexts, including ischemia–reperfusion injury [18] and diabetic kidney disease [19], in which SIRT1 activation is generally protective through mechanisms involving mitochondrial homeostasis and oxidative stress modulation [20]. These pathological settings share important features with SA-AKI, particularly excessive ROS production, impaired mitochondrial energetics, and disrupted tubular function. Our findings are broadly consistent with this literature and extend it to experimental SA-AKI by showing that sepsis is accompanied by reduced renal SIRT1 abundance together with multiple indices of mitochondrial dysfunction.

Mechanistically, recent advances highlight several interconnected pathways through which SIRT1 may preserve mitochondrial function in SA-AKI. One prominent mechanism involves mitochondrial quality control *via* autophagy/mitophagy. Prior work has shown that SIRT1 activation can attenuate sepsis-induced AKI by promoting autophagy through Beclin1 deacetylation, thereby facilitating clearance of damaged mitochondria and limiting ROS amplification [21]. Although autophagy flux was not directly assessed in our study, the marked improvement in mitochondrial ultrastructure and membrane potential with SIRT1 activation is compatible with enhanced mitochondrial quality control and reduced accumulation of dysfunctional mitochondria. In parallel, the NAD^+^-SIRT1 axis has gained increasing attention in sepsis biology. Sepsis is associated with NAD^+^ depletion and dysregulated NAD^+^-dependent signaling, which can suppress SIRT1 activity and impair downstream mitochondrial programs. SIRT1 interacts with metabolic regulators such as PGC-1α to promote mitochondrial biogenesis and oxidative metabolism, supporting adaptive energy production under stress conditions [22].

Oxidative stress represents a central node linking sepsis to mitochondrial dysfunction and tubular injury [23]. Our data demonstrated increased ROS production and lipid peroxidation together with reduced antioxidant capacity under septic conditions, consistent with current understanding of SA-AKI as a redox-imbalanced state. Importantly, SIRT1 activation significantly reduced ROS and MDA levels and partially restored antioxidant activity. SIRT1 has also been reported to regulate inflammatory signaling, including NF-κB-related pathways [24]. However, inflammatory cytokines and downstream inflammatory signaling were not measured in the present study. Accordingly, although our findings are compatible with an anti-inflammatory role of SIRT1 in septic renal injury, this aspect remains inferential and should be addressed directly in future work.

A strength of our study is the combined assessment of mitochondrial structure, mitochondrial function, and energy utilization. In SA-AKI, impaired tubular transport often reflects functional metabolic shutdown. Therefore, ATPase activity provides a physiologically meaningful indicator of bioenergetic competence. Na^+^/K^+^-ATPase and Ca^2+^-handling ATPases are major ATP consumers required for epithelial polarity, ion homeostasis, and tubular reabsorptive function. Suppression of ATPase activity is a hallmark of tubular energetic failure and contributes directly to epithelial dysfunction. In both cell and animal models, septic stress reduced ATPase activity, and SIRT1 activation partially reversed these changes. These results suggest that SIRT1 related signaling may contribute not only to mitochondrial integrity but also to maintenance of energy-dependent tubular function under septic conditions. Notably, certain systemic or global indices such as ATP content or NEFA levels may show only modest, non-significant trends *in vivo* at a single early time point, likely reflecting biological variability and compensatory metabolic routing in CLP-induced sepsis [25].

Lipid metabolic disturbance has also emerged as an important contributor to metabolic imbalance in SA-AKI. In the present study, NEFA levels tended to increase after CLP surgery and showed a decreasing trend after SRT1720 treatment; however, these changes did not reach statistical significance. Therefore, our data do not provide definitive evidence that SIRT1 activation significantly alters circulating NEFA levels in this model. Further studies with larger sample sizes are needed to clarify whether SIRT1 activation affects lipid uptake, fatty acid oxidation, or systemic lipid redistribution during sepsis-associated renal injury. In addition, NAD^+^-boosting strategies have been reported to improve mitochondrial and inflammatory endpoints in kidney injury models [26], supporting the translational relevance of targeting NAD^+^/SIRT signaling in renal stress states [27].

From a translational perspective, pharmacological activation of SIRT1 has been explored in human inflammatory settings. A human endotoxemia study reported that selective SIRT1 activation reduced cytokine release and coagulation activation, suggesting that SIRT1-centered interventions may be feasible and biologically active in systemic inflammation [28]. Moreover, recent work in infectious models suggests that SIRT1 activation can improve immunometabolic regulation and outcomes [29]. Although translation to SA-AKI requires caution, these findings collectively support the plausibility that SIRT1 represents a therapeutically tractable node at the interface of metabolism, mitochondrial function, and inflammation.

In conclusion, this study demonstrates that SIRT1 expression is significantly reduced in SA-AKI and that modulation of SIRT1 activity markedly influences mitochondrial structure, oxidative stress burden, energy metabolism, and renal injury severity. By preserving mitochondrial integrity, restoring membrane potential, limiting oxidative stress, and maintaining ATP-dependent enzymatic function, SIRT1 activation exerts a protective role in septic renal injury. These findings suggest that targeting SIRT1-mediated mitochondrial energy homeostasis may represent a promising therapeutic strategy for SA-AKI.

## Conclusion

In conclusion, this study demonstrates that septic stress is associated with reduced renal SIRT1 expression, mitochondrial dysfunction, impaired energy metabolism, and aggravated kidney injury in experimental models of SA-AKI. Pharmacological activation of SIRT1 alleviated renal injury and improved mitochondrial-related indices, including mitochondrial ultrastructure, oxidative stress, membrane potential, and ATP-dependent enzymatic function, whereas pharmacological inhibition of SIRT1 generally produced opposite effects. These findings suggest that SIRT1-related signaling may contribute to the maintenance of renal mitochondrial energy homeostasis during sepsis and may represent a potential therapeutic target for SA-AKI.

## Data Availability

All data generated or analyzed during this study are included in this published article. Additional data are available from the corresponding author upon reasonable request.
